# Transnational labour migration and the politics of care in the Southeast Asian family

**DOI:** 10.1016/j.geoforum.2011.12.006

**Published:** 2012-06

**Authors:** Lan Anh Hoang, Brenda S.A. Yeoh, Anna Marie Wattie

**Affiliations:** a School of Social and Political Sciences, The University of Melbourne, Melbourne, VIC 3010, Australia; b Department of Geography and Asia Research Institute, National University of Singapore, 1 Arts Link, Kent Ridge, Singapore 117570, Singapore; c Center for Population and Policy Studies, Gadjah Mada University, Bulaksumur G-7, Yogyakarta, Indonesia

**Keywords:** Transnational labour migration, Left-behind children, Family, Care, Southeast Asia

## Abstract

Recent increases in female labour migration in and from Asia have triggered a surge of interest in how the absence of the mother and wife for extended periods of time affects the left-behind family, particularly children, in labour-sending countries. While migration studies in the region have shown that the extended family, especially female relatives, is often called on for support in childcare during the mother’s absence it is not yet clear how childcare arrangements are made. Drawing on in-depth interviews with non-parent carers of left-behind children in Indonesia and Vietnam, the paper aims to unveil complexities and nuances around care in the context of transnational labour migration. In so doing it draws attention to the enduring influence of social norms on the organisation of family life when women are increasingly drawn into the global labour market. By contrasting a predominantly patrilineal East Asian family structure in Vietnam with what is often understood as a bilateral South-East Asian family structure in Indonesia, the paper seeks to provide interesting comparative insights into the adaptive strategies that the transnational family pursues in order to cope with the reproductive vacuum left behind by the migrant mother.

## Highlights

► We investigate care arrangements for children in Vietnamese and Indonesian migrant families. ► Surrogate carers in sending countries are the most disadvantaged in global care chains. ► Care relationships reflect prevailing power hierarchies and norms. ► The study signifies the need to account for local specificities in researching care in the South.

## 1 Introduction

Home to 60% of the world’s population, Asia has been the most important site of transnational migration in the past decades. Much of transnational mobility in and from Asia takes the form of short-term contract labour migration, which traces its origins to the contract coolie system of the colonial era (Massey et al., 1998, p. 181). The majority of Asian migrant workers are in the prime of their reproductive lives, leaving families including children behind in home countries. Although no accurate statistics are available, it is believed that tens of millions of children around the world are being left behind by their migrant parents, mostly lower-skilled workers from developing countries (Yeoh and Lam, 2006, p. 6). Migrant workers at the lower end of the socio-economic spectrum often have little or no possibility of family reunification in destination countries, resulting in their long-term separation from left-behind family members. Concerns about how parental migration affects left-behind children are further heightened by increasing numbers of female migrant workers on the move. The gender segmentation of the global labour market results in the clustering of female workers in personal and service sectors, particularly in care work and domestic work. In these “global care chains”^1^ (Hochschild, 2000), we witness transfers of care within and between families in both the North and the South with mixed and complicated outcomes for individuals involved.

In this paper we focus on the Southern end of global care chains, particularly on how migrant families in source countries adjust and negotiate care arrangements for left-behind children to accommodate the absence of parents, especially mothers, due to transnational labour migration. This is in part a response to the disproportionate attention to the Northern aspect of migratory movements in the literature on global care chains while little has been done on how migration affects care regimes in the South. As noted by Zimmerman et al. (2006, p. 19), “we know very little about how care deficits are addressed in sending communities and kin networks.” Moreover, the scanty literature on care in source countries is mostly concerned with perspectives of migrants as exemplified by the cluster of work on long-distance parenting (cf. Hondagneu-Sotelo and Avila, 1997; Parrenas, 2001a,b, 2008; Dreby, 2006) and, to a lesser extent, children themselves (cf. SMC, 2004; Parrenas, 2005; Dreby, 2007). In this paper we turn our attention to surrogate carers (i.e. kin members who provide surrogate care) whose voices have been largely absent from migration research. In so doing, we reemphasise the critical role of the extended family in the migration process – a fact that has been observed in different contexts (cf. Richter, 1996; Dreby, 2010; Hoang et al., 2010). Our focus is placed on migrant families’ decision making about care arrangements from the angle of non-parent carers in Indonesia and Vietnam. Northern Vietnam with its predominantly patrilineal family structure and Java in Indonesia with a more flexible bilateral one provide contrasting case studies for this paper, in view of our assumption that the choices made by transnational families, and the degrees of latitude they have in making these choices, are directly linked to differences in family structure. As suggested by Kofman and Raghuram (2009, p. 19), the analysis of care arrangements in the South needs to be attuned to local specificities.

This paper is not another attempt to confirm the relatively well-established fact that female relatives, and not fathers, often step into take over mothers’ care duties when the latter migrate. Rather, it aims to unfold the decision making process about care arrangements for left-behind children through the perspective of non-parent carers. Analyses in the paper centre on three main questions: (1) is the decision to turn to someone in the extended family for help with childcare merely pragmatic or driven by certain social norms and expectations (certain individuals are deemed fit or appropriate to fill the mother’s shoes)?; (2) What motivates surrogate carers’ acceptance of these care duties?; and (3) How do negotiations around care, if any, take place? Through the case studies of Indonesia and Vietnam, we establish that the organisation of care work in the Southeast Asian transnational family is deeply embedded in family norms and structures.

## 2 Care and migration in Asia

Care is defined by Standing (2001, p. 17) as the act of seeing to a person’s “physical, psychological, emotional and developmental needs.” It embraces a range of human experiences and relationships of obligation, trust, loyalty and commitment concerned with the well-being of others (Graham, 1983). Theories about the organisation of care have seen important developments, shifting from the concept of “care triangle” (i.e. state, market and family) (Esping-Andersen, 1999) to the “care diamond” which consists of state, market, family and community (Evers, 1996; Kofman and Raghuram, 2009). This conceptualisation of care is, nevertheless, not always relevant to developing countries in Asia where contributions of state, market and even community are weak or even non-existent. Asian care regimes remain strongly ‘familistic’, that is, families have the principal responsibility for their members’ welfare, be it in terms of sharing incomes or providing care to those in need (Esping-Andersen, 2000, p. 5).

Furthermore, Western-centric theorisations of care are implicitly premised on the conception of the household as a nuclear unit while responsibilities for care in less developed societies often extend to relatives beyond the nuclear household. Even in modern Asian societies with advanced economies like Singapore, Taiwan, South Korea and Thailand, it is still common for relatives to take care of the children during the day (when parents are at work) (Ochiai, 2009, p. 63). The extended family, as mentioned earlier, is particularly important in the context of transnational labour migration. For example, we have seen how male migration from Sri Lanka leads to the merging of nuclear households in a context where societal norms disapprove of women living ‘alone’ (Bruijn et al., 1992) and how female migration in the Philippines and Sri Lanka results in the expansion of the nuclear household when female relatives move into help fathers with childcare (Gamburd, 2000; SMC, 2004; Parrenas, 2005). In the Philippines, Indonesia and Thailand, the extended family has been found to play a major role in all aspects of migration including participating in the migration decision, funding the trip and taking care of left-behind children, thereby mitigating the social costs of migration (Hugo, 1995; Bryant, 2005).

Unsurprisingly, concerns about care in migrant families are mainly associated with the absence of the mother, and not the father. The so-called “crisis of care” (Parrenas, 2005, p. 12) or “care drain” (Hochschild, 2002, p. 17) only hits the family when the woman migrates. This is because care involves not only physical labour (‘caring for’) but also emotional labour (‘caring about’), (Hooyman and Gonyea, 1995; Lynch and McLaughlin, 1995), which is particularly important for children in their early developmental stages, a role for which the mother more than anyone else (or sometimes no one else) is deemed fit.^2^ Even if the left-behind father is willing to take over his wife’s care duties during her absence, he is still considered incapable of fully substituting for her. Indeed, research in different Asian countries has shown that children of migrant fathers fare better than those left behind by migrant mothers (Battistella and Conaco, 1998; SMC, 2004; Gamburd, 2005; Jampaklay, 2006). It has been assumed that the biologically different reproductive functions of men and women automatically construct differences in parenting behaviours (Silverstein and Auerbach, 1999, p. 2). Although this essentialist approach has been criticised as oversimplifying mothering and fathering (cf. Marsiglio and Pleck, 2005, p. 252), gender is still widely perceived as affecting parental behaviour since men and women possess different kinds of human capital and personal characteristics.

The consensus among migration studies in Asia is that it is the norms regarding gendered division of labour, rather than gendered differences in care skills and practices, that influence the organisation of care work in migrant families (Bruijn et al., 1992; Gamburd, 2000; Hugo, 2002; SMC, 2004; Parrenas, 2005). While male migration is rarely disruptive to the family since the mother simply continues her traditionally ascribed role of a carer and a nurturer in the father’s absence, female migration, on the contrary, requires significant adjustments on the part of the left-behind family. Despite some evidence of men’s greater involvement in childcare and domestic work when women migrate, the woman-carer model appears resilient as evident in the fact that many left-behind families turn to female relatives for help with caring tasks vacated by absent mothers. A survey of 1200 mother migrant households in Sri Lanka, for example, found that grandmothers made up 50% of primary carers, fathers – 26% and the rest – other female kin (Save_The_Children, 2006). In Central America and Mexico, women’s number one preference is for grandmothers to be caregivers during their absence (Hondagneu-Sotelo and Avila, 1997, p. 559; Dreby, 2010). The mobilisation of female relatives in care work has often been linked to left-behind fathers’ reluctance to assume care responsibilities because of the continued pressure to follow gender norms with respect to caring practices (Gamburd, 2000; Parrenas, 2005, p. 92). Female labour migration, therefore, appears to uphold rather than refract the conventional gender division of labour in the family.

## 3 Transnational labour migration from Indonesia and Vietnam

The history and scale of transnational labour migration from Vietnam and Indonesia differ greatly. Transnational migration is long established in Indonesia while it is a recent (but rapidly growing) phenomenon in Vietnam. Indonesia started labour exports in the 1970s and by mid-2006, there were 2.7 million Indonesians working overseas with official permission, representing 2.8% of the total national workforce (Hugo, 2007). Almost 700,000 Indonesians were deployed overseas in 2007 (Sukamdi, 2008, p. 326). In fact, it is believed that Indonesia could surpass the Philippines to become the largest labour-sending country in Asia if undocumented flows, especially migration to Malaysia, are accounted for in the statistics (Wickramasekera, 2002, p. 13). In 2006, according to Malaysia’s Ministry of Home Affairs, 1.3 million documented workers plus 70% of the estimated 700,000 illegal migrants in the country were Indonesian nationals (Hernández-Coss et al., 2008; Kanapathy, 2008).

Middle East and Malaysia are major destinations for Indonesian migrants although Hong Kong, Singapore and Taiwan are becoming increasingly important. The Middle East-bound flow is dominated by female domestic workers migrating on short-term fixed contracts leaving families behind while migration to Malaysia involves mostly men who may be accompanied by their wives and are likely to settle down permanently at the destination at some stage (Hugo, 1995, p. 282). In 2004, the proportion of Indonesian female workers in Malaysia was 49% while women accounted for 94%^3^ of registered Indonesian workers in Saudi Arabia. The number of Indonesian female labour migrants started to grow larger than male labour migrants in the 1980s and reached 80% in 2008.^4^ In 2005, more than a million Indonesian female migrants were employed as domestic workers in the Middle East and Asia (Hugo, 2007).

The volume of Vietnam’s labour exports has been increasing rapidly since the early 1990s when the country switched its target from the collapsed communist bloc in Eastern Europe (which was Vietnamese workers’ main destination in the previous two decades) to other markets. Vietnam had 500,000 overseas workers in 2008 with remittances totalling USD 1.6 billion annually (Dang et al., 2010, p. 12). During 2002–2007, around 70,000 Vietnamese workers left the country for work overseas each year and the Vietnamese government aims to achieve a total of one million nationals working abroad every year starting 2015 (PANO_Economy, 2007; Nguyen, 2009, p. 1). Vietnamese workers are now present in over 40 countries with Malaysia, Taiwan, South Korea and Japan being the main destinations. Migration from Vietnam is still largely dominated by males despite the steady increase of female migrant workers in the past decade – women accounted for 33.3% in 2008 (Dang et al., 2010, p. 13).

## 4 The CHAMPSEA study

**Fig. 1:**
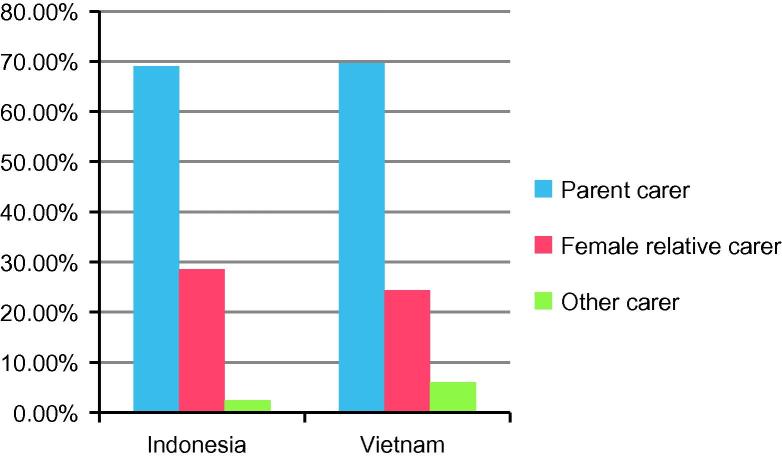
Carers for left-behind children in migrant families in Indonesia and Vietnam.

**Fig. 2:**
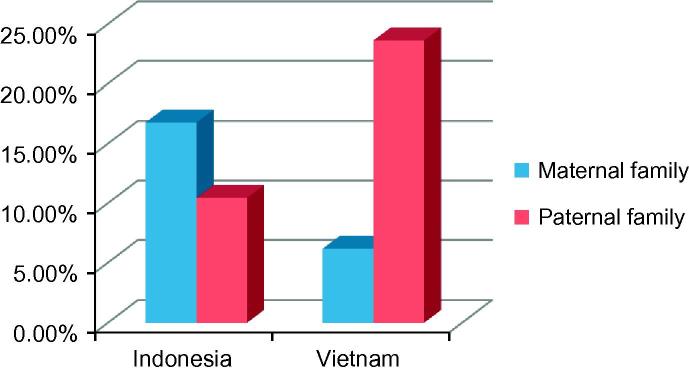
Non-parent carers of left-behind children in migrant families by family line.

The paper builds on a multi-method cross-sectional research programme^5^ (also known as CHAMPSEA) that investigates impacts of parental transnational labour migration on the health and well-being of left-behind children in four Southeast Asian countries – Indonesia, the Philippines, Thailand and Vietnam. The CHAMPSEA Study collected both quantitative (around 1000 households in each country) and qualitative data (around 50 households in each country) from migrant and usually resident households. The total number of migrant households involved in the quantitative survey was 534 in Indonesia and 551 in Vietnam. Figs. 1 and 2 show the composition of non-parent carers’ group in our quantitative sample by gender and family line respectively. We can see in Fig. 1 that left-behind parents were most important as carers for children in migrant families, accounting for almost 70% in both Indonesia and Vietnam. The proportion of female relative carers was slightly higher in Indonesia (29%) than in Vietnam (24%), meaning there was a greater number of “other carers”^6^ in Vietnam – 33 cases (6%) – as compared to 13 cases (2.4%) in Indonesia. Vietnam was a unique case where grandfathers had a visible presence in the surrogate carers’ group (*n* = 32) while they were almost negligible in the other three countries (*n* = 2, 4, and 1 in Indonesia, Philippines and Thailand respectively). What should be noted is that only one-fifth of grandfather carers in Vietnam were from the maternal side of the family, the rest were paternal grandfathers.

The importance of the paternal family as surrogate carers for children in Vietnam becomes more pronounced in Fig. 2 – paternal family carers accounted for nearly 24% of the Vietnam sample and maternal family carers – 6%. Interestingly, the picture obtained from Indonesia is different – there were more surrogate carers from the maternal side although the difference between maternal and paternal families was not significant (16.8% and 10.4% respectively). The figures suggest that there are important dynamics underlying differences in care arrangements in the two countries that require further investigation.

This study primarily draws on in-depth interviews with 18 non-parent carers of left-behind children – 10 in Indonesia and eight in Vietnam.^7^ The fieldwork was conducted in East Java in Indonesia and Thai Binh Province in Vietnam, both with relatively high incidence of transnational labour migration in the respective country. Again, the qualitative study sample shows a difference in family structure between the two countries – seven out of ten carers in Indonesia were on the maternal side whereas all but one carers in Vietnam belonged to the paternal family. Most carers were grandmothers (15 out of 18 cases) while the rest included two ‘other female relative carers’ in Indonesia and a paternal grandfather in Vietnam. Most Indonesian carers were not working during the time of the fieldwork whereas all Vietnamese carers were engaged in income-generating activities of different sorts despite the fact that the latter were slightly older than the former (the mean age was 56 and 58.6 for Indonesia and Vietnam respectively). In terms of migration status, most households were mother-migrant (*n* = 11) or both-parent-migrant (*n* = 6) and the only father-migrant case was in Vietnam with the mother working full-time in a nearby factory.

## 5 The carer – care as a matter of altruism, obligation and reciprocity

The differences in care arrangements between Vietnam and Indonesia discussed earlier prompted us to delve into the qualitative data for possible explanations. In particular, we look at carers’ responses to this question: ‘*How and why was it decided that you took care of [TARGET CHILD NAME]*?’ Due to the open nature of the interview, the question triggered diverse responses yet still revealed commonalities within and between the two study sites. From non-parent carers’ narratives emerged three important kinds of feelings that motivated their involvement in caring for children of migrant parent(s) – altruism, a sense of obligation, and the expectation of reciprocity.

The grandmother was automatically the first choice as surrogate carer when both parents migrated although the norm of which family line (maternal versus paternal) she belonged to differed between the two countries. When the grandmother was unavailable, one of the female relatives would be called on for help. In many cases in both Indonesia and Vietnam, decision making about care arrangements for left-behind children hardly took place at all, especially if the carer was involved in childcare before migration. The rather simple transfer of care in Indonesia was possibly facilitated by the fact that the carers, all of whom were female, were not working before and after the child’s parents migrated. Only in a few Indonesian cases did the carer indicate that the care arrangement initially conflicted with her personal plan, which nevertheless did not stop the child’s parents from migrating overseas. Nasimi, who is quoted below, was one of them:

‘They entrusted him to me, they asked me to take care of him, that’s all... because there was no one else. His grandmother was old and she was taking care of Angin’s older brother... She lives over there... The other grandparents live far from here... I was asked: *“Nasimi*,^8^*do you mind taking care of Angin?”* I was surprised by their request... I still wanted to work at that time. They said: “*This is my turn to earn a living for my children, to pay for their school tuition*.” Then I agreed.’

Nasimi – Angin’s mother’s sister – was in fact a transnational labour migrant herself who had migrated to Brunei Darussalam to do domestic work three times. She took leave in Indonesia every 2 years between contracts and that was when Angin’s parents asked her to take care of him so that they could migrate to Saudi Arabia. Angin, who was only 2 months old when his parents first left him with his aunt, has since grown emotionally attached to her and her husband. Though she still wanted to re-migrate at the point of Angin’s parents’ departure, Nasimi did not turn down their request. She added happily at the end of the interview: ‘*I had always wanted to have a son and then Angin was entrusted to me*!’

Nasimi was not alone as Budini – a 65-year-old maternal grandmother – had also planned to migrate to Saudi Arabia ‘*to be a maid*’ when her daughter decided to leave her 10-year-old daughter behind with Budini to migrate to Hong Kong. Apart from Nasimi and Budini, all Indonesian carers in our study did not report any conflict of interest, or in fact any extensive need for deliberation, in taking over care duties from the child’s migrant parent(s) when approached by them. For grandmothers, helping their children with childcare so that they could migrate overseas for work was a natural thing. Mirah – a 61-year-old maternal grandmother – for example, was amused by our questions on how and why it was decided that she took care of her 3-year-old grandson Dwija so that his mother could migrate to Hong Kong to work as a domestic worker. Her only response when approached by her daughter was: ‘*I will take good care of him when you are away*.’ Similarly, Niaidy – a 50-year-old maternal grandmother – told us that there was no discussion of any sort about care arrangements for the then 2-year-old Cinta when his mother migrated to Taiwan. In these cases, the migration decision was mainly discussed between the migrant and his/her spouse while the carer’s consent to provide surrogate childcare was often taken for granted. In not any single instance was childcare arrangement reported to be problematic. Our findings re-emphasise the Javanese strong moral obligation for relatives to take care of unattended family members, particularly children and the elderly. It is related to the value of *rukun*, a Javanese idea of living in harmony which in practice refers to the absence of overt interpersonal conflict and requires mutual attention and assistance among family members and neighbours in time of needs (Geertz, 1961).

The passive role of surrogate carers in decision making about childcare was also observed in Vietnam, albeit to a lesser extent. Tan – a paternal grandmother – for example, told us that her son neither consulted her about his migration to Malaysia nor requested her help in looking after his 3-year-old daughter – Thom – despite sharing the house with her:

‘He just left without saying a word to me about that (care for his daughter)... His wife couldn’t have asked anyone else to help as her parents had passed away. I have always been looking after the girl like this...’

Thom’s mother, who did not get along with Tan, was working 26 days a month as a seamstress in a nearby factory on 12-h shifts and thus totally dependent on Tan with regard to childcare. Her working hours were so strict that she could not even take a day off when Thom was ill and hospitalised. At the time of our fieldwork, the relationship between Tan and her daughter-in-law was so tense that they refused to speak to each other despite living in the same house. Her son maintained regular phone communication with home but he only spoke to his wife each time he got in touch. Yet, the grandmother did not mind looking after Thom because ‘*she is my son’s child*’ and felt happy with her care responsibilities even though ‘*her parents don’t say a word to me or give me a thing*.’ Tan made it clear that she was not expecting any rewards in return.

Research has already shown that, regardless of migration status, households in ‘developing’ Asia rely heavily on the extended family for support with childcare (Chen et al., 2000; Kofman and Raghuram, 2009, p. 16; Ochiai, 2009). The involvement of the extended family, particularly grandparents, in childcare even before migration makes care arrangements for left-behind children less problematic. In many Vietnam cases where decision making about care arrangements was reportedly simple and straightforward, there was in fact no transfer of care from the migrant parent(s) to the surrogate carer because the latter had been involved in caring for the child before migration. Migration was, therefore, neither unsettling nor upsetting for both the carer and the child under study. In non-parent carers’ narratives we felt a mixed sense of obligation and altruism underlying their involvement in childcare notwithstanding the extra economic hardship it brought. These feelings are best summed up in Giang’s – a paternal grandmother – words:

‘Did I have another choice? Her parents were struggling economically so I had to support them – they are my son and daughter-in-law. I agreed (to look after my granddaughter) without any hesitation. Her father is my son.’

Altruism was manifest in the general lack of financial incentives for carers. There was hardly any concrete financial arrangement made at the point of the migrant’s departure. Although many, but not all, interviewed surrogate carers received regular allowances to cover the child’s expenses, rarely were they given any remuneration for childcare except small gifts, mainly in the form of medicine. Exceptions included an Indonesian maternal grandmother who said that the remittances intended to cover their child’s expenses helped improve her own diet^9^ and another maternal grandmother (both in Indonesia^10^) who received 100,000 rupiah (about USD 11) a month from the migrant mother. Son – a Vietnamese maternal grandmother – paid for all her grandchildren’s expenses because their parents had incurred large debts to fund their migration:

“I use my own money to pay for their expenses because their parents haven’t earned enough to settle the debt. It will take them at least one or 2 years to repay it in full... Even if they give me money, I wouldn’t take any... They are still in debt now... They have not earned enough to settle the debt so there’s no reason I should receive money from them.”

In our Indonesia sample, migrant mothers often remitted money to left-behind fathers who might or might not pass on some of it to carers to cover the child’s expenses. In the Vietnamese families, on the contrary, many migrant mothers remitted money to their natal family even when fathers and paternal family members were main carers of children. It was, therefore, common to find surrogate carers paying parts or all of the child’s expenses from their own pockets. Except one Vietnamese paternal grandmother,^11^ who recorded all expenses of the child in a notebook so that her parents could pay back later, not any carers openly expected that their contributions would be returned or rewarded in the future. This relates to research elsewhere in the region that finds grandparents’ involvement in childcare without any financial support pervasive in Asian poor working families regardless of migration status (Kofman and Raghuram, 2009, p. 16).

It is, however, premature to conclude that all surrogate carers looked after left-behind children out of pure altruism. In Vietnam and Indonesia where social welfare for the elderly, particularly those living in rural areas, is extremely limited,^12^ children are the main source of support and care for the elderly. Indeed, some carers like Cilik below expected that their children would look after them in their old age in return for their help with childcare during migration:

‘My son (the migrant father) takes care of me with his whole heart. My daughters are busy with their own business. His younger brother never finished washing my laundry…never finished... It’s true, he (the migrant father) is the one who takes care of me. If I am not patient with my granddaughter, who will take care of me in my old days?’

On the same note, Nyaidi said that she was happy to take care of her 4-year-old grandson because ‘*when he gets older*, *I can ask him for help easily*.’ Similar expectations were also expressed by Su – a paternal grandmother in Vietnam – ‘*I tell them (migrant parents) that when I get too old to look after myself, they’ll have to take care of me*.’ Also, she was eager to help her son and daughter-in-law with childcare because thanks to her they could work to earn money and ‘*If they have some money, I won’t have to worry about them or support them in the future.*’ Clearly, taking care of grandchildren was more than just an obligation in these cases. Rather, it was carers’ investment strategy for the future. These expectations of reciprocity were, nevertheless, rarely articulated explicitly between carers and migrant parents but simply assumed by carers themselves when agreeing to take over the latter’s care duties.

## 6 The left-behind father – looking at the usual “scapegoat” from the carer’s angle

Whilst migrant women have been hailed as “modern-day heroines” in some Asian countries (e.g. Parrenas, 2001a,b, p. 1136), the men they leave behind are often portrayed in a negative light. In the Philippines and Sri Lanka, for example, research shows that men tend to reject care duties vacated by the migrant mother because the reversal of gender roles is seen as a threat to their masculinity (Gamburd, 2000; Parrenas, 2005). Some studies even suggest that rather than taking up the responsibility for care giving, men appear to need care when women migrate (Kofman and Raghuram, 2009, p. 12). As a result, men left behind in the wake of women’s transnational labour migration tend to emerge as truants from the realm of the left-behind family which is somewhat contradicted by what we found in Vietnam. In fact, in contrast to what has been observed in Sri Lanka and the Philippines that female relatives make up the majority of carers for left-behind children when the mother migrates (Parrenas, 2005; Save_The_Children, 2006), our quantitative survey found that left-behind fathers constituted the largest group of principal carers for their children – 67% and 71% in Indonesia and Vietnam respectively. Although survey questions may have elicited normative answers, there are reasons to believe that these figures are not erroneous. First, the nucleation of the family in both countries in recent decades (cf. Hugo, 1995, p. 275; Nguyen, 2003, p. 76; Wahyuni, 2005, p. 3) has made the family more self-reliant than ever resulting in lesser involvement of the extended family in both productive and reproductive spheres. Furthermore, with significantly reduced fertility rates and with it, smaller family sizes, in Indonesia and Vietnam,^13^ greater investment is made towards the child’s development, particularly with regard to education and discipline, making the family prudent in deciding who to leave the child with when a parent migrates.

These survey findings are supported by what we found in qualitative interviews – left-behind fathers did not shirk their parental duties as commonly believed even when they were not recorded as principal carers of children. This reminds us of Gamburd’s (2000) observation in Sri Lanka that there is actually more male participation in childcare and domestic work than reported. Most left-behind fathers who were less involved in caring for the child (as compared to the surrogate carers) and hence not reported as main carers were holding full-time jobs or taking care of other children in the family. Whilst it was common for left-behind mothers to cut down or even cease her participation in paid work to take care of children when fathers migrated, many men were unwilling to do so for fear of compromising their masculine identity, which is inextricably attached to the economic provider’s role (Hoang and Yeoh, 2011). In Vietnam, all but one left-behind father, who was in the rehabilitation centre for drug addiction at the time of the fieldwork, were working full-time in the absence of the mother and the children were thus in the care of a relative. The Indonesian fathers who were economically inactive were caring for the target child’s siblings or other relatives. Despite these other commitments, most of them were still involved in caring for the TC in their limited time off work – cooking and feeding the children, bathing them and washing their clothes, supervising and monitoring their studies in the evening. Farul’s father in Indonesia, for example, left her with her paternal grandmother when he was busy with farm work but took her back into his care during slack periods. Similarly, Dwija’s farmer-father and maternal grandmother took turns to take care of him. Although Mirah – Dwija’s grandmother – was recorded as his main carer, she told us during the interview that actually care work was equally split between her and the boy’s father. In Vietnam, we also found a similar case where Tho – a 10-year-old girl stayed with her long-distance merchant father when he was home but moved over to her paternal grandparents’ house when he was away.

A common arrangement in both countries was that children stayed with grandparents during the day and went back to their fathers at night so that the fathers could help them do homework. Notwithstanding their limited involvement in physical care work, the father in these cases remained the decision maker in matters concerning the child such as health care and education. More importantly, they were in charge of disciplining the children and supervising their studies, which grandparents were often considered incapable of. Similar to what has been observed in Mexico (cf. Dreby, 2010, p. 158), grandparents were perceived as more indulgent than parents in caring for children and more lenient towards them when it came to disciplining. The left-behind father’s role in childcare, according to the carers we interviewed, was important despite the limited time they put into it.

## 7 Family structures and the politics of care

In this section of the paper we seek to understand why children were left with one side of the family (maternal/paternal) and not the other and explore what led parents to arrive at particular childcare arrangements. Left-behind children are often depicted in migration literature as passive recipients of care and lacking agency in migration processes (Parrenas, 2005; Dreby, 2007). This appears to be the case in our study as well, for reasons embedded in the existing context of care. Since many non-parent carers had been looking after the child since before his/her parent migrated overseas, their continued involvement in childcare after migration appeared unproblematic because the child had grown emotionally attached to the surrogate carer over time. For this reason the child appeared passive in care arrangements which was, nevertheless, not the case in the event post-migration transfer of care conflicted with his/her preference – s/he would actively seek to undo the decision. This is supported by Orellana et al.’s (2001, p. 578) argument that children’s agency may take particular twists when their relations with adults are situated in transnational social fields. Four-year-old Cinta in Indonesia, for example, refused to live with her father despite his insistence and insisted to live with her maternal grandparents instead. In another situation, 11-year-old Faris was sent to his paternal grandparents to live after his parents left but he returned to his maternal grandparents after a month. According to Sarmi – Faris’ maternal grandmother – he wanted to return to her because he did not “*feel at home*” at his paternal family’s place:

“Actually he even went to school there. But it didn’t even last a month before he returned here... He didn’t like to be there, he told us... He didn’t feel at home there... in fact...his grandfather there is a *kamituwa*^14^...he is used to be here all the time... He doesn’t feel at home there.... since he was born here, so he wants to... he always requested to come home here... after 1 day or 2 days... he already requested to come back here...”

Unlike the patrilocal^15^ Vietnam, new families in Java, Indonesia are more likely to be established in proximity to the bride’s extended family, which partly explains the maternal family’s greater involvement in childcare before and after migration in our study site. Wahyuni (2005, p. 14) has also noted the importance of the maternal family in providing care for children left behind by internal migrant parents in Java. In the context of a bilateral kinship system in Indonesia (cf. Geertz, 1961), there are no rigid rules pointing to a particular family side as the more powerful in the kinship hierarchy. Decisions about childcare in the Indonesian migrant family were, therefore, more likely to be pragmatic than political – children were cared for by someone they liked to live with or who was available and willing to take care of them – although the maternal family tended to be a more popular choice. The reasons provided by Indonesian carers for assuming care responsibilities for left-behind children, as opposed to passing the responsibilities to the other side of the family, were mostly straightforward: “her grandmother over there is already taking care of many (grand)children”, “her house is too far from places”, or “they are too old (to take care of the child).”^16^

Similarly, in half of Vietnam cases we were told that a particular care arrangement was made because grandparents on the other side of the family were unavailable due to old age, death, poor health, economic hardship or other care commitments. However, while the Indonesian carers mentioned above came from both sides of the family, all these four Vietnamese carers were paternal grandmothers. They had been living next door to or in the same house with the child’s family prior to the parents’ migration and had thus been more involved in childcare. Yet, this is not to say that the decision to leave children with the paternal family was simply a practical issue of paternal relatives’ continued involvement in childcare. As illustrated by the story of Huyen below, patrilineal norms made the transfer of care responsibilities to the maternal family an extremely sensitive issue. Huyen, a 78-year-old paternal grandfather, took care of 10-year-old Dien and his 12-year-old sister while their mother was working in Taiwan. Dien’s nuclear family had been sharing the house with Huyen since his parents got married while his maternal grandparents were living in the same village. Huyen was entirely dependent on his meagre income from farming to sustain livelihood expenses and yet received no remittances from Dien’s mother. Dien’s father became addicted to drugs soon after his mother left for Taiwan, stole neighbours’ assets to feed his addiction and was sent to the rehabilitation centre by force after Huyen made a written plea to the local authorities. Dien’s mother had been working in Taiwan for 4 years and had no plan to return home in the near future. She first went there to work for a company but left her contracted job after a year to seek work “outside” which presumably paid her better.

Saddled with childcare responsibilities and financial hardship, Huyen had repeatedly suggested to his daughter-in-law, both directly and indirectly, that the children should be sent over to their maternal grandparents who were better-off. Neither Dien’s mother nor her natal family objected to this request but the maternal grandparents would take over chilcare only on the condition that Huyen took the children to their house to “*hand them over*”, which Huyen refused to do: “*How on earth would a paternal grandfather send his own grandchildren to their maternal grandfather’s care?*”

Huyen’s difficult situation was compounded by Dien’s refusal to move into his maternal family’s home as suggested by his grandfather even though their house was closer to his school. Dien was determined in making his own choices:

“I told Dien to go to his maternal grandparents’ place to have lunch after the morning class because his (maternal) grandfather has a motorbike to take him back to school (for the afternoon class). Otherwise, it would be hard for him to walk back here. He did that for a few days but he has been coming home in the past few days. I said to him: “I will not cook for you if you don’t go there.” He replied: “I will cook for myself if you don’t.”

Political issues over care arrangements for Dien and his sister were complicated by both the estrangement between his drug-addicted father and his migrant mother and the frosty relationship between the two sides of the family. In the Vietnamese patrilineal tradition, grandchildren belong to the paternal family (Rydstrom and Drummond, 2004, p. 9) who, as a result, commands authority in matters concerning them. Care arrangements in our Vietnam study, therefore, tended to reflect family power hierarchies rather than practical concerns which do not necessarily work to the advantage of the paternal family. As in the case of Huyen, childcare duties exacerbated his economic hardship because Dien’s mother sent all her remittances to her natal family, not providing him with any financial assistance. When asked why he did not ask his daughter-in-law for money to cover the children’s expenses, Huyen replied: ‘*I feel I should not do so. They are my grandchildren*.’ Clearly, as a paternal grandfather, Huyen felt that taking care of his grandchildren was not a ‘favour’ he did for his son but an obligation that he could not reject. The children’s maternal grandparents who were much better off, on the other hand, could afford not to care for them or give them any material support without incurring social criticism because this was not their primary obligation.

Huyen’s case was not atypical in our Vietnam sample. Narratives referring to patrilineal norms as the basis for care arrangements can be found in many other interviews. Su – a 58-year-old paternal grandmother, for example, provided us with an intriguing account of how care was arranged for her granddaughter when the child’s mother was leaving for Malaysia:

‘At the beginning she told me that she would ask her mother to take care of the girl. She took Hoan’s clothes to her mother’s house. But then the maternal grandmother brought the clothes back to me and asked me to look after Hoan, saying that she couldn’t look after her... She might have thought that, as Hoan was my son’s daughter, I should have been the one to look after her.’

Similarly, Giang, also a paternal grandmother, repeatedly emphasised throughout the interview that she was taking care of her granddaughter because ‘*her father is my son*.’ The embeddedness of care arrangements in the Vietnamese patrilineal tradition is crystallised in Vu’s words:

“It is common that the father’s family should always rank first, before the mother’s. It wouldn’t sound alright if the kids’ parents send them to their maternal grandparents before approaching their father’s family. We wouldn’t mind but people would gossip if their parents ask their mother’s family for help. They would wonder what’s wrong with the paternal family. Most of them would think in this way.”

Vu’s grandchildren, in fact, preferred to live with their maternal grandparents. She told us that the children would go straight to their maternal grandparents’ place after school if no one from the paternal family picked them up. Their maternal grandparents, according to Vu, would have been very happy had the children lived with them because “*the kids can warm up their house*” but “*they politely hide their feelings*” out of respect for the paternal family who should have priority over the children.

## 8 Conclusion

That the extended left-behind family, despite their immobility, has a critical role to play in transnational labour migration is not new. The migration scholarship in Asia, however, has been mostly concerned with gender norms and practices underlying fathers’ reluctance to assume care duties left behind by migrant mothers while surrogate carers’ perspectives are largely unknown. We have pointed out that feelings and thoughts, health and wellbeing of surrogate carers in labour-sending countries have been largely marginalised even though they are often the most vulnerable and disadvantaged of all in global care chains. In global care chains, as noted by Hochschild (2000, p. 136), poorer women raise children for wealthier women while still poorer – or older or more rural – women raise their children. For this reason, global care chains both reflect a basic inequality of access to material resources and reinforce global inequalities by redistributing care resources from the poor to the less poor and from the less poor to the rich (Yeates, 2004, p. 373). In other words, care refracts and reproduces existing social hierarchies (Kofman and Raghuram, 2009, p. 18). In our study countries, as most surrogate carers are elderly grandparents who need care themselves, a more important question arises: “Who cares for the carers?” It opens up an avenue for researching the interplay of care, migration and class/inequalities in developing countries.

Foucault (1980, pp. 97–98) argued that every human relationship is a struggle and negotiation of power and that subjects are materially constituted by power relations and always part of them. Care relationships at the Northern end of global care chains are characterised by inequalities in terms of wealth, race, gender and class (Hochschild, 2000, 2002; Yeates, 2004). In the South, issues of power and inequality are less salient due to the extensive involvement of family and kinship networks in the provision of care.^17^ Kofman and Raghuram (2009, p. 7) suggest that care in the Southern context is broadly seen to be provided through some mix of altruism, social contract and reciprocity, which is supported by our findings in Indonesia and Vietnam. More importantly, through the case of Vietnam we establish that care relationships in the transnational family also reflect the prevailing power hierarchies and norms which accord a certain kinship group with more rights as well responsibilities towards children. Differences in care arrangements between Indonesia and Vietnam as a result of differing familial structures (bilateral versus patrilineal) clearly demonstrate the need for more flexible frameworks for researching care in the South that not only account for local specificities but also consider the wider socio-cultural context in which the family is embedded.

## Footnotes

1 “Global care chains” are defined by Hochschild (2000, p. 131), as “a series of personal links between people across the globe based on the paid or unpaid work of caring.”

2 The woman-carer model, however, does not apply to all Asian societies. Men in Minangkabau ethnic group in Indonesia, for example, are known to shoulder the bulk of care duties.

3 Fact Sheet – Migration, Remittance and Female Migrant Workers, http://siteresources.worldbank.org/INTINDONESIA/Resources/fact_sheet-migrant_workers_en_jan06.pdf, date accessed 28 September 2010.

4 Statistics on Indonesia Worker by Destination Country and Sex, Ministry of Manpower and Transmigration of the Republic of Indonesia, http://www.nakertrans.go.id/pusdatin.html,12,217,pnaker, date accessed 03 February 2009.

5 The CHAMPSEA Study was funded by The Wellcome Trust, UK and coordinated by the National University of Singapore (Singapore) and the University of St. Andrews (Scotland, UK). The authors of this article were involved in the collection of both quantitative and qualitative data in Indonesia and Vietnam in 2008 and 2009.

6 Other carers include brothers, uncles, grandfathers and domestic workers.

7 The rest of the qualitative study sample were parent-carer families.

8 Names of respondents have been changed to ensure anonymity.

9 Interview with Halimah, maternal grandmother, 67 years old.

10 Interview with Mirah, maternal grandmother, 61 years old.

11 Interview with Su, paternal grandmother, 58 years old.

12 Evans, Gough et al. (2007) analysis of the 2004 Viet Nam Household Living Standards Survey (VHLSS) shows that on average, social welfare is received by 14% of the elderly in Vietnam, pension – 18% and that almost 67% of the Vietnamese elderly receive no regular formal transfers.

13 The fertility rates of Indonesia and Vietnam were 2.6 and 2.5 respectively in 2007 (Source: AMC 2008, Asian Demographic and Human Capital Data Sheet 2008, www.populationasia.org).

14 A Javanese term for head of the village.

15 Patrilocality – the coresidence of married couples with the husband’s parents – remains a pervasive feature in Vietnamese society – a survey conducted in 1995 found that 75% of the married respondents had lived with the groom’s family after marriage (Hirschman and Nguyen, 2002).

16 Interviews with Rukiyah (paternal grandmother, 60 years old), Halimah (maternal grandmother, 67 years old), and Mirah (maternal grandmother, 61 years old).

17 It was still rare for migrant families in our study countries to hire domestic workers to take care of left-behind children. Only eight out of 2114 migrant households we surveyed in Indonesia, the Philippines, Thailand and Vietnam hired domestic workers during migration – seven cases were in the Philippines and one in Indonesia.
